# One Health policy for combatting African trypanocide resistance

**DOI:** 10.1016/j.onehlt.2024.100871

**Published:** 2024-08-05

**Authors:** Keneth Iceland Kasozi, Ewan Thomas MacLeod, Susan Christina Welburn

**Affiliations:** a Infection Medicine, Deanery of Biomedical Sciences, Edinburgh Medical School, College of Medicine and Veterinary Medicine, The University of Edinburgh, 1 George Square, Edinburgh EH8 9JZ, United Kingdom; b School of Medicine, Kabale University, Kabale University, Box 317, Kabale, Uganda; c Key Laboratory of Tropical Translational Medicine of Ministry of Education, The School of Tropical Medicine, The First Affiliated Hospital, Hainan Medical University, Haikou 571199, Hainan, People's Republic of China; d School of Global Health, Chinese Centre for Global Tropical Disease Research, Shanghai Jiao Tong University School of Medicine, Shanghai 200025, People's Republic of China; e Zhejiang University - University of Edinburgh Joint Institute, Zhejiang University, International Campus, 718 East Haizhou Road, Haining 314400, People's Republic of China

**Keywords:** Neglected tropical diseases, One Health, COVID-19, Trypanosomiasis, Developing countries, Global health, Funding, Infectious diseases

## Abstract

The rise of African trypanocide resistance (ATr) is influenced by various factors such as evolutionary changes in the pathogen, the presence of resistance genes in the population, poor policy decisions, limited private-public partnerships to engage local communities, and insufficient funding for the development of new drugs over the past sixty years. These challenges have been exacerbated by the inadequate implementation of drug liberalization policies in the mid 20th century, leading to poor pharmacovigilance practices for veterinary drugs in low and middle income countries (LMICs). One health (OH), a disease management framework, provides practical solutions for addressing ATr, drawing on its success in managing previous epidemics like avian influenza in 2004 and the recent COVID-19 pandemic, where institutional collaborations were rapidly established. To combat ATr, OH initiatives involving both international and local partners at the policy and grassroots levels are crucial to generate community interest. The importance of political commitment, media involvement, and nongovernmental organizations cannot be overstated, as they are essential for resource mobilization and long-term sustainability in LMICs.

## 1 Introduction

There is increasing scientific evidence for the evolution of antimicrobial drug resistance (AMR) to the drugs available to treat human African trypanosomes (HAT) and animal African trypanosomiasis (AAT) [1,2]. In most rural communities, inappropriate use of trypanocides is considered to confer selection pressure for resistance genes to develop in trypanosome populations [3]. The emergence of drug resistance phenotypes has complicated both control AAT and impeded progress towards the elimination of HAT as a public health problem by 2030 [4,5]. Drug resistant trypanosomes have been found in humans [6], livestock [7] and the tsetse fly insect vector [8] and this is a key concern due to weak regulatory policies contributing to the growing antimicrobial resistance (AMR) in endemic countries. Given the emergence of drug resistance and lack of new trypanocides in development for AAT, the use of chemotherapy alone as a primary method to control AAT in sub–Saharan Africa is no longer a sustainable approach [9].

The fear of influenza pandemics was instrumental in galvanizing international attention and collaborative efforts which led to the birth of one-health (OH). The global anxiety surrounding the highly pathogenic avian influenza (HPAI) subtype H5N1, commonly known as bird flu in 2004 was driven by its high mortality rate in poultry and the potential for the virus to mutate and spread to humans, causing a severe and widespread outbreak [10]. This provided an opportunity to unify the Food and Agriculture Organization (FAO), World Organization for Animal Health (WOAH), World Health Organization (WHO) and the World Bank and develop comprehensive strategies to address emerging zoonotic diseases [11,12]. By fostering collaborations, these agencies pool their expertise and resources to develop more effective strategies to address zoonotic diseases. Interdisciplinary collaborations bring together experts from various fields, including veterinarians, epidemiologists, environmental scientists, public health professionals, and economists. This creates a complex web while developing comprehensive solutions. Furthermore, international, and national authorities having equal partnership demonstrates a need for a strong leadership platform. The threat of HPAI H5N1 did recede (A. L. [13]), however, COVID-19 demonstrated the importance of having a strong operational OH policy in place [14]. Furthermore, OH in rabies control has been associated with a drop in the number of cases and helped veterinarians gain more confidence in handling cases, reduced production costs and increased sharing of information between veterinarians and medical sectors [15], despite the limited rabies surveillance systems which are scarce in Africa [16]. In Latin America, the commitment and engagement of the health sector have been pivotal in integrating rabies control into broader national rabies control programs. This integration has significantly contributed to the successful implementation of OH principles in the region [17]. COVID-19 exposed the dysconnectivity between governmental promotion of OH and the reality outside of governmental agencies [18,19]. OH creates a platform for leadership and knowledge sharing, as exemplified by the Canadian OH platform which has members from the political sciences, sociology, law, economics, anthropology, veterinary, human health, public health, environmental sciences, epidemiology and implantation research [20]. In line with the WHO post COVID-19 preparedness plan for the next pandemic (Tedros [21]), OH offers a feasible platform to address the growing African trypanocide resistance challenge on the African continent. In essence, by addressing diseases in animal populations, we not only protect the health and welfare of animals but also contribute to the broader goals of ecosystem health, biodiversity conservation, and sustainable resource management. This holistic approach is crucial for maintaining healthy and resilient ecosystems in the face of global challenges.

### 1.1 Liberalization of the African trypanocide industry

Demands to open the African market/industry for drug manufacture and supply have led to the establishment of local manufacturing pharmaceutical companies with linkages to multinational European counterparts. Severe economic pressures and declining manufacturing in the late 1970s were associated with the birth of the liberalization policy which included the pharmaceutical industry in several African countries [22]. For effective prevention of AMR development, national regulatory frameworks are required, ideally established by thorough intergovernmental collaborations on OH. These help develop and promote good manufacturing practice (GMP) regulations regionally and enable harmonized monitoring strategies [23]; GMP remains a significant challenge in most low and middle income countries (LIMICs) and the requirement for more active collaboration in drug monitoring and guidance in both human and animal health ministries is long overdue. The World Trade Organization (WTO), WHO, and WOAH are all committed to collaborate more closely to create better infection control strategies [24], but too often communication channels operating at the ministerial level are not translated to grassroot administrative units, making it challenging to implement OH messaging and good practice in these communities.

To address HAT resistance (HATr), collaborative international partners have supported biomedical research into novel therapies such as nifurtimox/eflornithine combinations. Drugs have been repurposed and clinical trials are in advanced stages. In contrast, resources for new drug options for AAT resistance (AATr) are lacking, arising from disproportionate allocation of funding in the WHO/WOAH related biomedical and clinical research [25]. This situation has been worsened further during the COVID-19 pandemic where disproportionate funding has seen increased poverty and marginalization for the control of NTDs in LMICs [26]. Ignoring investment in new drugs and appropriate existing drug use in livestock poses a significant risk to human and animal health, and this is very important in LMICs where veterinary policy is weak (A. L. [27]). The WHO 2021–2030 road map has been updated for the elimination of HAT and AAT as a public health problem [28] which is an update from the previous target were *g*HAT was a target for transmission alone [4]. This implied WHO prioritized (*gambiense*) *g*HAT over (*rhodesiense*) *r*HAT (see [29] where new drugs for *g*HAT are elaborated) and with these new developments, it is apparent *Trypanosoma brucei* species are now a priority. Attempting to control a zoonotic infection without long-term management of infection in reservoir species is unlikely to be successful. Chronic failures to address AATr also contribute to human spillover infections (HATr) [3]. AATr control is complicated by the weak veterinary policies (poor surveillance, diagnostics and political commitment) in endemic communities [30,31], where net migration (re-stocking and livestock trade) contributes to the re-introduction of infections in human populations (A. L. [27,32]). There was already a funding crisis for tackling neglected tropical diseases (NTDs) [33,34,35], exacerbated by the impacts of COVID-19 and the difficult decisions that needed to be made in the allocation of budgets. Given that some NTDs are widening their range as a result of climate change, travel and trade [36], and considering lessons learned from the COVID-19 pandemic [37], a lack of investment will stifle the WHO/FAO trypanosomiasis elimination target. Taking a more holistic OH approach to infection control can reap significant benefits (A. [38]). For example, OH in East Africa (Uganda) in the control of emerging and re-emerging zoonotic infections i.e., Ebola, Marburg, plague, avian influenza, Rift valley fever, anthrax and yellow fever [39] as well as NTDs, especially on HAT [40] and the strengthening of national laboratories to detect zoonotic infections [41] do provide evidence for the benefits of multidisciplinary-sectoral collaborations. A comprehensive list of successful stories for the control of NTDs through an integration of OH [42] generates momentum for the adoption of OH for the management of ATr in the post pandemic age.

### 1.2 African trypanocide therapeutics in the COVID-19 age

The *Stamp Out Sleeping Sickness* (SOS) campaign [43], in Uganda whereby insecticides combined with trypanocides administered in cattle contributed to the reduction of the HAT burden is a classical example were OH has been successful. SOS involved multidisciplinary international collaborative initiative for the control of an NTD in Eastern Africa. Despite challenges associated with the implementation of OH as exemplified through the SOS project under the Coordinating Office for Control of Trypanosomiasis in Uganda (COCTU) such as leadership, ownership, and limited long-term financial support which demonstrated the importance of financial resource allocation to resolve conflicts (A. L. [44]), the COCTU secretariat did receive political endorsement and local support thus making COCTU one of the most successful global OH bodies now being reabsorbed into the Ministry of Health (Uganda) in the post-pandemic era. Another successful case study where OH has been successful involved the rabies campaign in developing countries. In Tanzania during the rabies elimination project funded under the Bill and Melinda Gates Foundation, engagement with several key ministries and the Prime Minister's Office helped to generate momentum for project ownership despite infrastructure challenges due to cold chain for vaccine handling, and road access (A. L. [44]). This provides evidence that OH remains relevant today to address AMR in Neglected Tropical Diseases (NTDs) including challenges of counterfeits that are proliferative in most developing countries.

Inadequate, excessive or substandard antimicrobial drugs contribute to treatment failures, allowing infections to persist and potentially become resistant to available treatments [45]. This situation poses a grave threat to public health, especially in regions where access to quality healthcare and effective medications is already limited [46]. However, across rural communities in sub-Saharan Africa, farmers continue to buy substandard and unregulated trypanocides, for use in their animals, many not licenced in the market into which they are being sold [47] simply because they are cheap, and do not observe safe practice withdrawal periods prior to selling their animal given there is no monitoring in place [48]. From the community perspective, it is inevitable that a smallholder farmer in the absence of professional advice will opt for cheap drugs regardless of quality, but farmer-based treatments characterized by poor dosing, and failure to observe withdrawal periods do raise major public health risks as well as major animal welfare concerns. Concerns as to drug administration by farmers are not limited to trypanocides, and in countries with poor veterinary and human drug regulatory policies, as is common in the global south, lax regulations, infrastructure challenges, and a chronic lack of legal oversight create an environment where AMR can develop, unimpeded. Adoption of a holistic OH approach to combat AMR provides real opportunities for national governments with these infrastructure challenges to maximize the available human resources [49].

The interconnectedness of health as demonstrated by the COVID-19 pandemic and its consequences justifies the social and economic benefits of amalgamating professionals to work under one administrative framework. Unfortunately, while informal memorandums of collaborations between institutions have been written in line with WHO recommendations [50], the adoption of OH has rarely been mainstreamed into Government policy. A multitude of resources have been made available by the WHO to support and guide nations for the institutionalization of OH. Furthermore, WHO has encouraged LMICs to develop collaborative initiatives in preparation for the next pandemic. Most high-income countries (HICs) e.g., USA and UK have developed their response plans as was observed during the early phases of the pandemic [51,52], however, LMICs are at risk of being left behind. Individualism among nations works contrary to the United Nations (UN) Collective Action Plan, however, the political atmosphere in UN member states is responsible for these divergent policy decisions which work against efforts to promote planetary health for the control of epidemics.

The success of the COVID-19 response was, in part, due to strong political leadership and coordinated efforts at various levels of government and international bodies. This was especially important to counteract strange conspiracy theories and denialism (see [53] in the UK, Ireland, Spain, USA and Mexico) thus helping bring diverse perspectives from politics, funders, healthcare and policymaking under one framework [54]. For AMR and NTDs, similar political commitment is crucial. Governments need to prioritize these issues, allocate resources, and support international collaborations. The media played a pivotal role in framing COVID-19 as a pressing global crisis, which drove public engagement and compliance with health measures [55]. To address AMR and NTDs, it is essential to harness media and communication strategies to raise awareness, educate the public, and advocate for urgent action. The pandemic showed the importance of global cooperation. For diseases affecting LMICs, creating and maintaining strong international partnerships among governments, non-governmental organizations, and private sectors can enhance resource mobilization, research, and intervention efforts.

The private sector played a role in developing COVID-19 vaccines and treatments [56], its involvement is critical in the fight against ATr. Engaging pharmaceutical companies, biotech firms, and other private entities can drive innovation and ensure the availability of effective treatments and diagnostics. The Bill and Melinda Gates Foundation through GALVmed approved over USD 19 million for phase 3 trials for new drugs against AAT [57], and more recently collaborations between GALVmed, the UK Government Foreign Commonwealth and Development Office, Boehringer Ingelheim and GALVmed have made a concerted effort to combat ATr [58]. Unfortunately, reciprocal collaborations at the grassroot movements involving LMIC institutions and the government remain scarce. This implies media mobilization to advocate for ATr control at the community level, though crucial remains elusive. Empowering local organizations and communities to participate in and lead health initiatives can improve the effectiveness and sustainability of ATr interventions.

## 2 Conclusion

Although the WHO, WOAH, WTO, FAO work together, oversight of OH activities remains idealistic especially at the national level. Individualism, protectionism, duplication of research, and corruption as well as a lack of a streamlined leadership framework are major barriers to the realization of the OH strategy for infection control. To improve institutional preparedness in developing countries in anticipation of the perfect pathogen (Pathogen X = infectious like COVID-19, and virulent like Ebola), WHO has promoted OH to reduce spillover events. Without clear national and regional policies on OH, this objective will remain elusory in both HICs and LMICs. A coordinated approach that integrates political will, media engagement, global collaboration, private sector involvement, and grassroots mobilization is essential for addressing ATr effectively. By learning from the COVID-19 response, stakeholders can work together to create a powerful, united front against African trypanosomiasis in LMICs.

## Author disclaimer

The views expressed in this publication are those of the author(s) and not necessarily those of the NIHR or the Department of Health and Social Care.

## Ethical approval statement

Not applicable.

## Funding source

This research was supported by the 10.13039/501100000272National Institute for Health Research (16/136/33) using United Kingdom aid from the United Kingdom Government (SCW). This work was also supported by 10.13039/501100004835Zhejiang University Research Fund (SCW) and funded by the 10.13039/501100000867Commonwealth Scholarship Commission (UGSC–2021–447) in the United Kingdom and the Small Grants Programme of the 10.13039/501100000683Royal Society of Tropical Medicine and Hygiene (2021) in partnership with the 10.13039/501100000272National Institute for Health Research (NIHR) (KIK).

## CRediT authorship contribution statement

**Keneth Iceland Kasozi:** Writing – review & editing, Writing – original draft, Visualization, Validation, Software, Resources, Methodology, Investigation, Funding acquisition, Conceptualization. **Ewan Thomas MacLeod:** Writing – review & editing, Validation, Supervision, Resources, Project administration, Methodology, Investigation, Funding acquisition, Formal analysis, Data curation. **Susan Christina Welburn:** Writing – review & editing, Validation, Supervision, Software, Resources, Project administration, Methodology, Investigation, Funding acquisition, Formal analysis, Data curation, Conceptualization.

## Declaration of competing interest

The remaining authors declare that the research was conducted in the absence of any commercial or financial relationships that could be construed as a potential conflict of interest.

## Data Availability

No data was used for the research described in the article.
